# AgroLD: a knowledge graph for the plant sciences

**DOI:** 10.1186/s12863-025-01359-6

**Published:** 2025-10-03

**Authors:** Pierre Larmande, Bertrand Pittolat, Ndomassi Tando, Yann Pomie, Bill Gates Happi Happi, Valentin Guignon, Manuel Ruiz

**Affiliations:** 1 DIADE, IRD, CIRAD, Univ. Montpellier, Ave Agropolis, Montpellier, France; 2 AGAP, CIRAD, INRAE, Univ. Montpellier, Ave Agropolis, Montpellier, France; 3 French Institute of Bioinformatics (IFB) - South Green Bioinformatics Platform, Bioversity, CIRAD, INRAE, IRD, F-34398 Montpellier, Montpellier, France; 4 Bioversity International, Bioversity, Parc Scientifique Agropolis II, Montpellier, France

**Keywords:** Knowledge graphs, FAIR, Linked data, Bioinformatics, Plant sciences

## Abstract

**Background:**

The demand for food is expected to grow substantially in the coming years. To address this challenge, especially in the context of climate change, a deeper understanding of genotype-phenotype relationships is crucial for improving crop yields. Recent advances in high-throughput technologies have transformed the landscape of plant science research. However, there is an urgent need to integrate and consolidate complementary data to understand the biological system.

**Results:**

We introduce AgroLD, a knowledge graph that uses Semantic Web technologies to seamlessly integrate plant science data. AgroLD is designed to facilitate hypothesis formulation and validation within the scientific community. With approximately 1.08 billion triples, it integrates and annotates data from more than 151 datasets across 19 distinct sources.

**Conclusion:**

The overarching goal is to provide a specialized knowledge platform addressing complex biological questions in the plant sciences, including gene participation in plant disease resistance and adaptive responses to climate change.

## Introduction

Agronomic research is witnessing an unprecedented revolution in the acquisition of various data, such as phenotypic and genomic data, as well as data related to the functional characterization of specific genes. Understanding the intricate interactions between genotypes and phenotypes that lead to particular traits is a key focal point within this field. However, these interactions are difficult to identify because they occur at different molecular levels in plants and are strongly influenced by environmental factors (i.e., climate change). The new challenges include identifying these interactions and spanning diverse molecular entities contributing to phenotypic expression. This endeavor necessitates a holistic approach, incorporating insights from different data stacks into a comprehensive model to unravel the true functioning of biological systems.

For researchers, navigating through vast amounts of dispersed information across multiple online databases—each with distinct data models, scales, and access modes—is a major challenge. This is particularly evident in genetic association studies such as genome–wide association studies (GWAS), which establish links between genomic regions (loci) and phenotypic traits. GWAS loci often encompass multiple genes, necessitating thorough analysis to identify relevant genes. A similar challenge exists in transcriptomic studies, where researchers must interpret extensive lists of differentially expressed genes and determine which genes merit further laboratory investigation. Inevitably, researchers must decide which genes warrant further investigation in the laboratory, a decision often on the basis of subjectivity and incomplete data reviews. Today’s significant challenges are related to developing methods to integrate these heterogeneous data and enrich biological knowledge. Scientists also need methods to explore this large amount of data and to highlight relevant information that can be used to identify key genes.

The Semantic Web introduces techniques and technologies to transform vast amounts of data into actionable knowledge. It is a fundamental component of the Findable, Accessible, Interoperable, and Re-usable (FAIR) principles [1] - by enhancing data interoperability. This achievement hinges on establishing standardized vocabularies and ontologies, which systematically capture domain knowledge and translate it into semantic resources, empowering computers to index, search, and reason over data. Notably, the Resource Description Framework (RDF) [2] has gained widespread utilization for web-based data publication, leading to the creation of the Web of Data. Recently, numerous initiatives have emerged within the biomedical and bioinformatics domains, each aiming to provide comprehensive platforms for building scientific hypotheses around gene functions, phenotypic expression, and disease emergence. Illustrative examples include Bio2RDF [3], UniProt RDF [4], PubChem [5] and WikiPathways [6]. In the domain of human biology, notable contributions have been made through the establishment of platforms such as the DisGeNET RDF [7] and the Monarch Initiative [8]. Similarly, the field of plant science has yielded the Knetminer [9], a graph database designed to unravel plant molecular networks for analogous objectives. In this context, AgroLD [10, 11] was developed with the ambition of providing the tools and methods needed to exploit the data and knowledge produced within the plant community. AgroLD has been actively developed. Currently, AgroLD contains more than 1,08 billion triples, resulting from the integration of approximately 151 datasets gathered in 33 named graphs.

## Methods

### Information content

AgroLD was designed to accommodate the molecular and phenotypic information available on various plant species with a large focus on tropical crops. Since the first release [10], 40 new species (6 since the previous release [11]) have been integrated, including cereals, legumes, and fruit trees. The list of the 51 species is available in Table 1.

**Table 1:** The 51 plant species integrated in AgroLD

Species name	Common name	Taxon ID
Aegilops tauschii subsp. strangulata	rough-spike hard grass	200361
Amborella trichopoda	Amborella	13333
Ananas comosus	pineapple	4615
Arabidopsis halleri subsp. gemmifera		63677
Arabidopsis lyrata subsp. lyrata	Cardaminopsis lyrata	81972
Arabidopsis thaliana	thale cress	3702
Beta vulgaris ssp. vulgaris	sugar beet	3555
Brachypodium distachyon	stiff brome	15368
Brassica napus	rape	3708
Brassica oleracea var. oleracea	wild cabbage	109376
Brassica rapa	field mustard	3711
Citrus x clementina	clementine	85681
Coffea canephora	robusta coffee	49390
Daucus carota subsp. sativus	carrot	79200
Digitaria exilis	White fonio	1010633
Glycine max	soybean	3847
Gossypium raimondii	Peruvian cotton	29730
Helianthus annuus	domesticated sunflower	4232
Hordeum vulgare subsp. vulgare	two-rowed barley	112509
Malus domestica	apple	3750
Manihot esculenta	cassava	3983
Musa acuminata subsp. malaccensis	wild Malaysian banana	214687
Nicotiana attenuata	wild tobacco	49451
Olea Europaea	Mediterranean olive tree	158383
Oryza barthii	African wild rice	65489
Oryza brachyantha	malo sina	4533
Oryza glaberrima	African rice	4538
Oryza glumipatula		40148
Oryza longistaminata	long-staminate rice	4528
Oryza meridionalis	Australian wild rice	40149
Oryza nivara		4536
Oryza punctata	red rice	4537
Oryza rufipogon	common wild rice	4529
Oryza sativa Indica Group	long-grained rice	39946
Oryza sativa Japonica Group	Japanese rice	39947
Phaseolus vulgaris	common bean	3885
Prunus avium	Sweet cherry	42229
Prunus dulcis	almond	3755
Prunus persica	peach	3760
Saccharum spontaneum	wild sugarcane	62335
Setaria italica	foxtail millet	4555
Solanum lycopersicum	tomato	4081
Solanum tuberosum	potato	4113
Sorghum bicolor	sorghum	4558
Theobroma cacao	cacao	3641
Triticum aestivum	bread wheat	4565
Triticum dicoccoides	wild emmer wheat	85692
Triticum turgidum subsp. durum	durum wheat	4567
Triticum urartu	red wild einkorn wheat	4572
Vitis vinifera	wine grape	29760
Zea may	maize	4577

AgroLD is built incrementally and spans many aspects of plant molecular interactions. Initially, it integrated information on genes, proteins, metabolic pathways, and genetic studies built from several resources such as Ensembl Plants [12], UniProtKB [4], Gene Ontology Annotation [13], Gramene [14], Oryzabase [15], RAP-DB [16], and MSU [17]. In its current version, AgroLD adds predictions of homologous genes from Ensembl Compara and biological networks from StringDB [18], RiceNetV2 [19], PlantTFDB [20], and PlantRegMap [21]. The size of the knowledge base has expanded by 16% since the last release [11], reaching 1.08 billion triples.

The biological community has guided the choice of these sources, as they are widely used and strongly impact the user’s confidence. We have also integrated resources developed by the local SouthGreen platform [22] such as TropGeneDB [23], a tropical plant genetics database; GreenPhylDB [24], a comparative genomics database for tropical plants; OryzaTagLine [25], a rice phenotype database and SniPlay [26], a rice genomic variation database. These resources combine experimental data from research groups in Montpellier and southern France. The online documentation provides an overview of the integrated data sources [27]. Table 2 provides an overview of the data sources integrated into AgroLD.

**Table 2:** Data sources integrated in AgroLD

Data sources	Nb of datasets	File format	Ontology used	Nb of triples
Oryzabase	2	TSV	GO,PO,TO	347 K
GO Associations	2	GAF	GO	6,440 K
Genome Hub	7	GFF	GO, SO	12,233 K
Gramene	6	Custom flat file	All	159 K
Ensembl	51	GFF	All	838,874 K
UniprotKB	2	Uniprot	GO, PO	60,034 K
Oryza Tag Line	2	Custom flat file	PO, TO, CO	282 K
TropGeneDB	2	Custom flat file	PO, TO, CO	20 K
GreenPhylDB	2	Custom flat file	GO, PO	3,627 K
SNiPlay	1	HapMap, VCF	GO	16,204 K
Q-TARO	2	TSV	PO, TO	20 K
MSU	2	Custom flat file	PO, TO	2,068 K
RiceNetDB	6	Custom flat file	PO, TO	5,879 K
StringDB	45	Custom flat file	GO	131,559 K
RapDB	3	GFF	PO, TO	1,026 K
PlantTftDB	12	Custom flat file	PO, TO	86 K
Interpro	1	Custom flat file	PO, TO	196 K
CEGResources	2	GFF	PO, TO	1,031 K
OBO ontologies	12	OWL		15,131 K
TOTAL	151			**1,077,303 K**

Ontologies are referenced as *GO* gene ontology, *PO* plant ontology, *TO* plant trait ontology, *EO* plant environnment ontology, *SO* sequence ontology, *CO* crop ontology (plant specific traits)

**Fig. 1:**
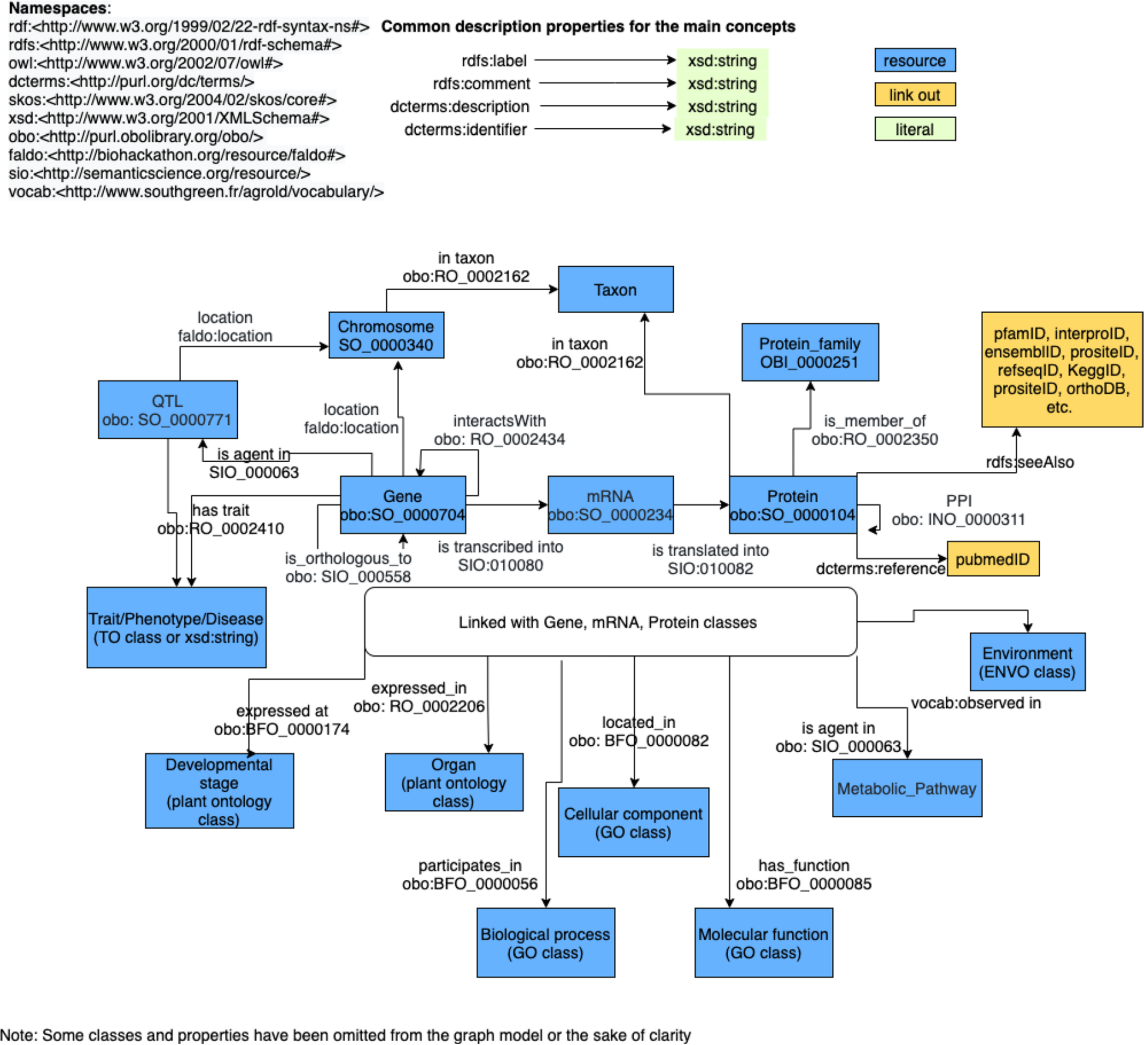
The AgroLD schema

We initially developed the conceptual framework of AgroLD on a custom vocabulary which also included mappings on well-established ontologies and controlled vocabularies in the fields of molecular biology and plant sciences such as Sequence Ontology [28], Gene Ontology [29], Plant Ontology [30] or Plant Trait Ontology [31]. Most of these ontologies are hosted by the Open Bio-Ontologies (OBO) Foundry project [32]. For this updated version, we modified the backbone schema (i.e., its vocabulary) by reusing other existing ontologies such as Semantic Science Ontology (SIO) [33], Feature Annotation Location Description Ontology (FALDO) [34], and Relation Ontology (RO) [35] to increase its interoperability with other knowledge graphs. Additionally, we included general ontologies such as Resource Description Framework Schema (RDFS), Simple Knowledge Organization System (SKOS), and Dublin Core to describe some properties of the biological entities. The online documentation shows the complete list of the ontologies used. Figure 1 shows a subset of the global schema of AgroLD [36].

### AgroLD integration pipelines

We developed various RDF conversion pipelines for large genomic and agronomic datasets. Although several generic tools exist within the Semantic Web community, such as Tarql [37], RML.io [38] or SPARQL-Generate [39] to name a few, none of them have been adapted to consider the complexity of data formats in the biological domain (e.g. Variant Call Format (VCF) [40]) or even the complexity of the information they could contain. A simple example illustrates this complexity through the Generic Feature Format (GFF) [41], which represents genomic data in a Tab Separated Value (TSV) format. It contains a column with a variable length key = value type information and different information depending on the data source. In this case, the transformation must be adapted according to the data source. Moreover, the large volume of data was a limiting factor for the abovementioned tools.

In this context, we developed RDF conversion tools adapted to various genomics data standards such as GFF, Gene Ontology Annotation File (GAF) [42], and VCF. Moreover, we are currently working on packaging these Extraction, Transform, and Load (ETL) tools in an Application Programming Interface (API) [43]. RDF conversion tools are Python-based scripts that can be run independently. Furthermore, the tools are tailored to run locally or use high-performance computing resources. More than forty scripts are available to process either data standards (e.g., GFF) or database-specific data (e.g., TAIR, RAPDB, and Oryzabase). Some parameters, such as the base Uniform Resource Identifier (URI), local paths, and RDF prefixes, can be defined globally. Parameters specific to a script can be defined at runtime. Documentation is available as a docstring for each script and explains how to run them. Moreover, the GitHub repository provides documentation on how to deploy and use the tools. Table 3 lists all the resources and tools available for AgroLD.

**Table 3:** Links to AgroLD resources and tools

Name of resource or tool and description, URL
Data
AgroLD datasets, https://doi.org/10.5281/zenodo.4694518
List of graphs, http://www.agrold.org/documentation.jsp
List of ontologies, http://www.agrold.org/documentation.jsp
AgroLD vocabulary, https://github.com/SouthGreenPlatform/AgroLD_ETL/tree/master/model
AgroLD SPARQL Endpoint, http://agrold.southgreen.fr/sparql
Example queries, http://www.agrold.org/sparqleditor.jsp
Tools
Web application, https://github.com/SouthGreenPlatform/AgroLD_webapp
RDF conversion pipelines (GFF2RDF, GAF2RDF, VCF2RDF, Datasets), https://github.com/SouthGreenPlatform/AgroLD_ETL

**Fig. 2:**
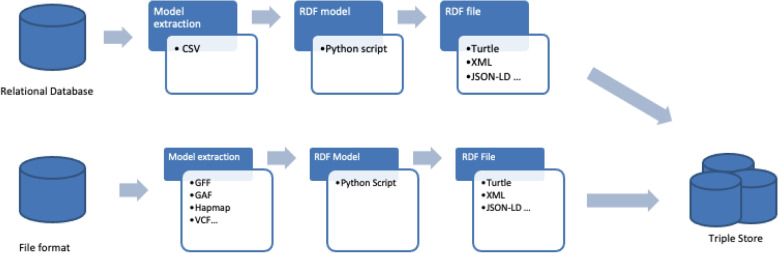
AgroLD ETL pipelines

To ensure that AgroLD remains updated with the latest data, the entire knowledge base is reconstructed annually. Additionally, new datasets are incorporated multiple times a year, typically every four months. Regularly updating the data presents challenges, as the original databases often lack automatic tracking of changes between versions. On the basis of our experience, completely reconstructing the knowledge base regularly is an effective strategy to bypass the complexities of handling data differences (Fig. 2).

### URI design and data linking

In the transformation pipelines, RDF graphs share a common namespace and are named according to the corresponding data sources. Entities in RDF graphs are linked by the common URI principle. We generally build URIs by referring to Identifiers.org [44], which provides design patterns for each registered source—for example, genes integrated from Ensembl Plants (http://identifiers.org/ensembl.plant/Entity_ID). When Identifiers.org does not provide them, new URIs are constructed, and in this case, URIs take the form (http://purl.agrold.org/resource/Entity_ID) In addition, the properties linking the entities are constructed in various forms (http://purl.agrold.org/vocabulary/property).

To link identical entities from different data sources, we used an approach based on URI pattern matching. Its principle is to scan the URIs to look for similar patterns in the terminal part of the URI (i.e., Entity_ID). In addition, we also follow the common URI approach, which recommends using the same URI pattern for two identical entities. Therefore, this allowed us to aggregate information from different RDF graphs for the same entity. In addition, we used cross-reference links by transforming them to URIs and linking the resource to the *rdfs* predicate seeAlso. This significantly increases the number of outbound links by reaching almost 80 million links, making AgroLD better integrated with other data sources. We plan to implement a similarity-based entity profile approach to identify matches between entities with different URIs.

## Results

To increase the accessibility of a broader user base, we developed a web application for AgroLD with multiple query interfaces. The initial interface facilitates keyword searches across the entire database content, enabling users to navigate the knowledge base. A more advanced search interface allows users to combine free text and apply filters on the basis of class types, properties, and external web services. This feature supports the aggregation of distributed data.

**Fig. 3:**
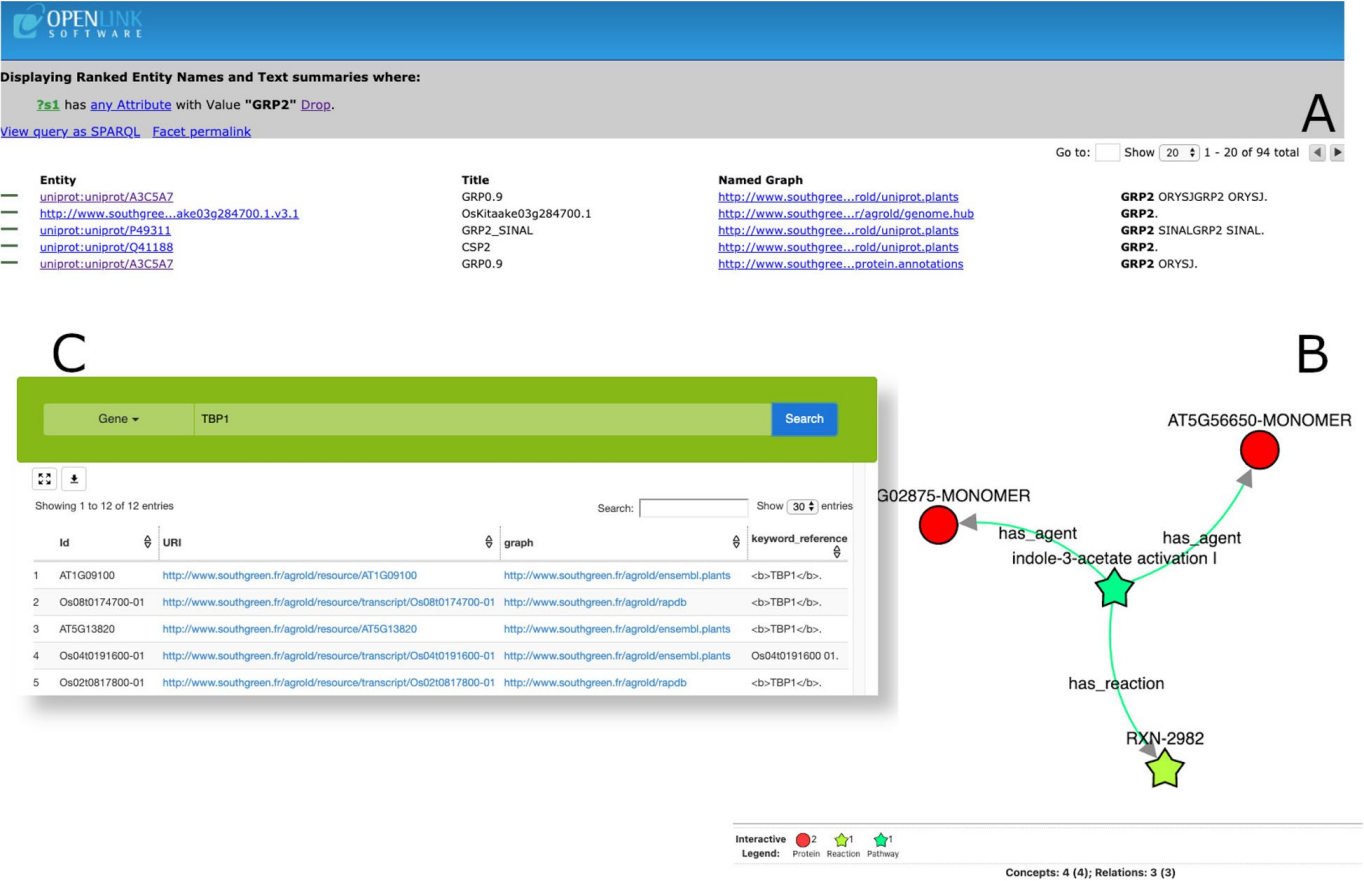
Overview of AgroLD Web interfaces. **A** displays the Faceted search interface. **B** displays results from the KnetMaps tool [45]. **C** displays results from the advanced search interface

**Fig. 4:**
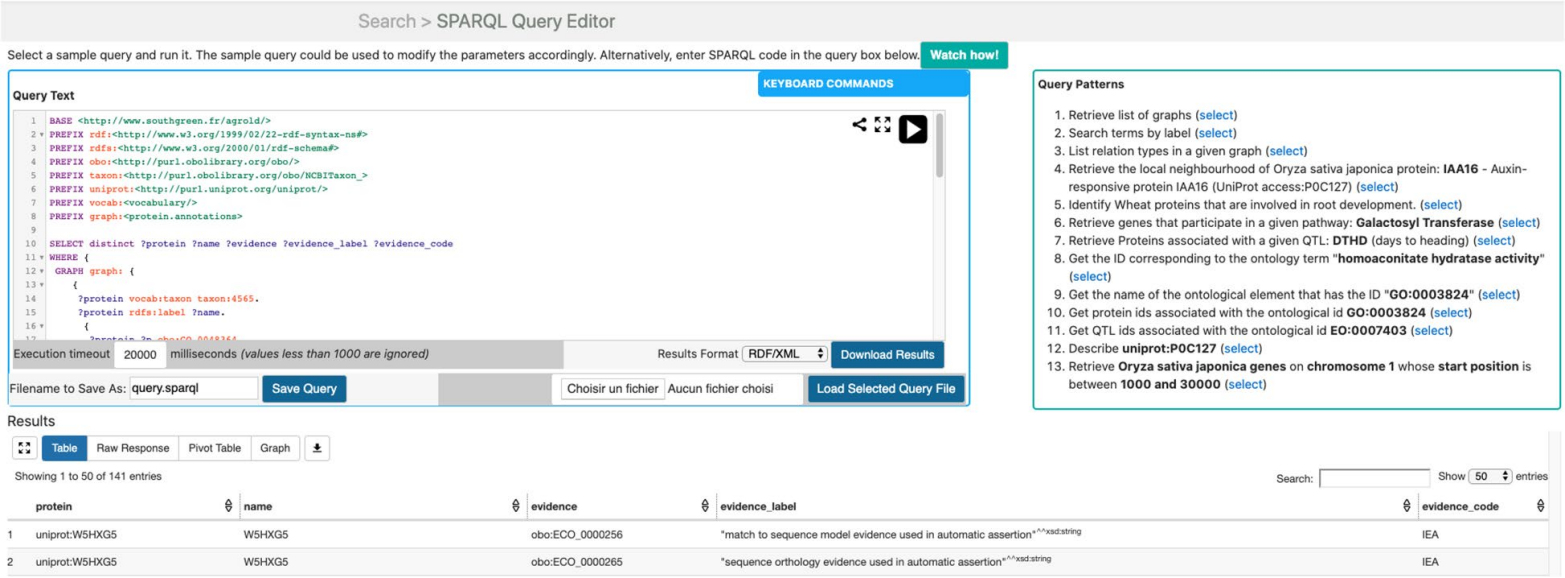
The SPARQL query editor. The Query patterns frame allows users to select a query from a natural language question. The Query text frame allows the visualization and modification of the SPARQL query. The results frame displays results returned from the query

We introduced a SPARQL Protocol and RDF Query Language (SPARQL) editor to address the challenge of handling SPARQL query language complexities, particularly for bioinformaticians and biologists. This editor provides an interactive tool for query formulation and result manipulation. Consequently, the AgroLD platform offers several entry points:

- ***Quick Search***: This plugin, powered by Virtuoso, uses faceted search capabilities to enable keyword-based searches and content navigation within AgroLD. Figure 3A illustrates the results of a keyword search, with *GRP2* used as an example. The results are ranked by the frequency of occurrence across various entity fields. The *Named Graph* column indicates the data source, whereas the *Title* and *Entity* columns display the entity names and their URIs, respectively. Clicking on a link provides a comprehensive view of the entity, and users can traverse entities via the provided HTTP links.
- ***Advanced Search***: This interface allows targeted searches on the basis of entity classes, incorporating an aggregation engine for external resources. Built upon a Representational State Transfer (REST) API (described below), the Advanced Search conceals the technical intricacies of SPARQL queries. The integration of the AgroLD API facilitates interactive searches across the knowledge base and external services such as Pubmed or EMBL. Figure 3C shows the user interaction: selecting the entity type (e.g., Gene) and providing keywords (e.g., TBP1) yield results presented in a sortable and downloadable table. Each row contains entity attributes, including the ID, data source, and context of the matching keywords. To obtain more details, users must click on the display link below the entity ID. This will open a new window (not shown). This window takes the name and description of the biological object in its header and then comprises several panels. Each of them shows one feature of the object displayed. They can differ according to the type of entity displayed (e.g. Proteins, Pathways, Publications, Terms associated, View as Graph, Expression, and See also panels). Figure 3B shows the View as Graph panel. It was adapted from Knetmaps [45]. It displays a window divided into two parts. On the left part, the entity is represented within a graph showing other entities linked to it (in this case, Pathways). More detailed information corresponding to the entity highlighted in green (not shown) is displayed on the right part. When the users select another entity in the graph, this content changes dynamically;
- ***AgroLD Restful API***: This programming interface supports interaction with the knowledge graph database. It comprises function calls grouped by entity classes within AgroLD (e.g., Genes, Proteins). For example, under the Gene class, functions exist for obtaining gene lists within genomic regions (genes/byLocus), genes matching specific keywords (genes/byKeyword), and genes encoding specific proteins (genes/encodingProtein).
- ***The SPARQL Editor***: We developed a SPARQL query editor with an interactive environment, employing YASQE and YASR tools [46] adapted for our system. The editor features modular and customizable query patterns aligned with user requirements. Figure 4 illustrates the editor’s layout, which is divided into three areas. The main area serves as the query field with syntax highlighting, error checking, autocompletion, and editing functions. Users can load and save queries and execute predefined query templates. The results appear beneath the editor, initially as a sortable table. JSON or graphical formats are also available for display and download.

## Discussion

The process of creating a knowledge graph is complex and challenging. In this section, we will present some of the challenges we had to address, particularly those related to managing the heterogeneity of the datasets and their sizes. We will discuss the challenges in aligning the entities and assessing the data quality.

With respect to data heterogeneity, the main problem was the variety of data formats, which we solved via RDF in a unified format. We propose several pipelines that can handle this variety and manage the dataset size. Indeed, as discussed in the pipelines section, in most cases, we preferred to develop our solutions rather than use generic tools to better manage the complexity or size of the datasets. Another problem is the heterogeneity of the genomic coordinates (i.e., different denominations of the chromosome identifier, missing information, etc.). We solve it by choosing a unique representation and transforming all coordinates into URI templates following the FALDO ontology representation [34].

With respect to the problem of entity linking (i.e., the same entities with different names or identifiers), we have only partially solved this problem, using pattern matching in URIs or database cross-linking to identify matches between entities. Indeed, in the case where the entities have a different namespace URIs (e.g., namespace1:identifier1 and namespace2:identifier1), we look for matching patterns in the URIs and create a new URI to establish the correspondence between them. If the entities have different URIs without matching patterns but with synonymous properties (i.e., skos:altLabel, skos:prefLabel, skos:synonym or specific properties), we look for matches with these properties and the patterns of the URIs. For entities that do not contain the above information, we take a more global approach based on property and value analysis. However, this is an open challenge that is currently being addressed.

With respect to the processes followed for data quality assessment, preprocessing quality assessments such as input file format, raw line, and missing value checking were developed for the resources used by the ETL pipeline. Next, the syntax of the triple products was validated via built-in libraries (e.g., with RDFlib). Other assessments include counting the number of entities (e.g., genes, proteins, chromosomes, etc.) and checking the presence/absence of properties with SPARQL query sets. More complex quality assessments, such as type restrictions on properties, are planned for the future.

## Conclusion

Data in the agronomic field are highly heterogeneous, multi-scale, and dispersed. For plant scientists to successfully address the challenges of their daily work, it is essential to integrate information on a global scale. Semantic Web technologies are central to data integration and knowledge management. The biomedical domain offers a good example to follow for capitalizing on previous experiences and considering the lessons learned. We have developed the AgroLD KG to leverage this approach in agronomy. AgroLD exploits the power of seamless data integration offered by RDF. It contains more than 1,08 billion triples, resulting from the integration of approximately 151 datasets gathered in 33 named graphs. However, the coverage of its species and data sources is expected to expand with subsequent releases. To our knowledge, AgroLD is one of the first initiatives to apply Semantic Web practices to the agronomic domain, playing a complementary role in the integrative approaches adopted by the community.

AgroLD is being actively developed on the basis of feedback from domain experts. It has also benefited from the support of the SouthGreen Bioinformatics Platform since its beginning in 2015 by providing IT support and infrastructure to host data and web applications. SouthGreen is one of the core platforms of the French Elixir-EU node and thus provides long-lasting support for AgroLD. AgroLD is strongly linked to several use-cases of the D2KAB (https://d2kab.mystrikingly.com) and DIG-AI projects (National Research Agency funded project) to demonstrate the benefits of linked data to discover gene-phenotype interactions. With the achievement of the current phase, user feedback reveals some limitations and challenges in the current version. Thus, several issues are a matter of ongoing or future work.

On the one hand, we must extend the KG coverage to more biological entities (e.g., miRNA, lncRNA, transposable elements) and relations (e.g., co-expression, regulation, and interaction networks) to capture a broader view of the molecular interactions. For example, we need to integrate information on gene expression and gene regulatory networks. On the other hand, the ETL process for KG creation is mostly based on domain-specific approaches, thus limiting its reusability. We will investigate approaches that use declarative functions for its creation.

Knowledge augmentation methods must be applied and adapted to the data. Indeed, we observed that some information remains hidden in the literal content of RDF, such as biological entities or relationships between them. Moreover, a large amount of related knowledge is available from external sources. We are currently developing methods to extract information embedded in unstructured data, such as KG text fields or external web documents and scientific publications, and bring this information in a structured form to the knowledge base. Finally, we extend the state-of-the-art data-linking techniques by considering the specificity of the biological domain.

## Data Availability

The AgroLD datasets can be found at the Zenodo repository https://doi.org/10.5281/zenodo.4694518. The Web application is available at https://github.com/SouthGreenPlatform/AgroLD_webapp and the RDF conversion pipelines (GFF2RDF, GAF2RDF, VCF2RDF, and datasets) are available at https://github.com/SouthGreenPlatform/AgroLD_ETL.

## Abbreviations

API: Application Programming Interface
ETL: Extraction, Transform, and Load
FAIR: Findable, Accessible, Interoperable, and Re-usable
FALDO: Feature Annotation Location Description Ontology
GAF: Gene Ontology Annotation File
GFF: Generic Feature Format
GWAS: Genome–Wide Association Studies
KG: Knowledge Graph
OBO: Open Bio-Ontologies
RDF: Resource Description Framework
RDFS: Resource Description Framework Schema
RO: Relation Ontology
REST: Representational State Transfer
SIO: Semantic Science Ontology
SKOS: Simple Knowledge Organization System
SPARQL: SPARQL Protocol and RDF Query Language
TSV: Tab Separated Value
URI: Uniform Resource Identifier
VCF: Variant Call Format
