# Correction: AgroLD: a knowledge graph for the plant sciences

**DOI:** 10.1186/s12863-026-01448-0

**Published:** 2026-06-11

**Authors:** Pierre Larmande, Bertrand Pittolat, Ndomassi Tando, Yann Pomie, Bill Gates Happi Happi, Valentin Guignon, Manuel Ruiz

**Affiliations:** 1 DIADE, IRD, CIRAD, Univ. Montpellier, Ave Agropolis, Montpellier, France; 2 AGAP, CIRAD, INRAE, Univ. Montpellier, Ave Agropolis, Montpellier, France; 3 French Institute of Bioinformatics (IFB) - South Green Bioinformatics Platform, Bioversity, CIRAD, INRAE, IRD, F-34398 Montpellier, Montpellier, France; 4 Bioversity International, Bioversity, Parc Scientifique Agropolis II, Montpellier, France

**Correction: BMC Genomic Data (2025) 26:73**

10.1186/s12863-025-01359-6

In the original version of this article, the given and family names of [Pierre Larmande, Bertrand Pittolat, Ndomassi Tando, Yann Pomie, Bill Gates Happi Happi, Valentin Guignon, Manuel Ruiz] were incorrectly structured. This has now been amended.

